# Neuroprotective synergy of vitamin D and exercise: a narrative review of preclinical and clinical evidence on aging-related neuroplasticity and cognitive health

**DOI:** 10.3389/fnut.2025.1642363

**Published:** 2025-09-09

**Authors:** Jingfeng Chen, Yang Li, Li Wang, Qi Liu, Bopeng Qiu

**Affiliations:** ^1^Department of Health and Physical Education, The Education University of Hong Kong, Hong Kong, China; ^2^College of Physical Education and Sports, Beijing Normal University, Beijing, China; ^3^Department of Physical Education and Sports, Shanghai Ocean University, Shanghai, China; ^4^Chinese Athletics Association, Dongcheng, Beijing, China; ^5^Antai College of Economics and Management Shangahi Jiaotong University, Xuhui, Shangahi, China; ^6^Shanghai Sports Science Society, Xuhui, Shangahi, China; ^7^School of Strength and Conditioning Training, Beijing Sport University, Beijing, China

**Keywords:** exercise training, vitamin D, cognitive function, neuroplasticity, aging

## Abstract

**Background:**

Both vitamin D and physical exercise have been independently associated with neuroprotective and anti-aging effects. However, their potential synergistic role in promoting healthy brain aging has not been fully clarified.

**Objective:**

This review examines the overlapping and potentially complementary effects of exercise and vitamin D on aging-related neurobiological and cognitive outcomes, with a focus on mechanisms relevant to older adults.

**Methods:**

We surveyed preclinical and clinical studies investigating the impact of vitamin D and exercise on neurotrophic signaling (e.g., BDNF, IGF-1), vascular and inflammatory pathways (e.g., VEGF, cytokines), and cognitive or functional outcomes in aging models and older human populations. Particular attention was given to recent randomized controlled trials (RCTs) such as SYNERGIC, DO-HEALTH, and PONDER, as well as large-scale epidemiological studies.

**Results:**

Preclinical findings suggest that vitamin D and exercise converge on shared biological pathways, including oxidative stress reduction, inflammation control, and neurogenesis promotion. Some animal studies demonstrated enhanced neuroprotection and cognitive improvement with combined interventions. In human trials, aerobic-resistance exercise with or without cognitive training improved cognitive scores in older adults with mild cognitive impairment, while vitamin D supplementation alone showed limited effect. Observational data further suggest that high serum vitamin D levels and regular physical activity are independently and jointly associated with delayed biological aging. However, evidence of clinically meaningful synergy remains limited, and no definitive conclusion can be drawn from current trials due to heterogeneity in design, population, and intervention protocols.

**Conclusion:**

While biologically plausible and supported by selective findings, the synergistic impact of vitamin D and exercise on brain aging has yet to be conclusively demonstrated in older adults. Future studies should focus on at-risk populations, standardized intervention models, and mechanistic outcomes to better evaluate their combined potential as low-cost, preventive strategies in aging.

## 1 Introduction

Aging is consistently accompanied by declines in memory, executive function, and processing speed, reflecting underlying impairments in neuroplasticity, the brain’s capacity to remodel synaptic connections and generate new neurons in response to experience (1). These changes are exacerbated in neurodegenerative disorders such as Alzheimer’s and Parkinson’s diseases, where disrupted neurogenesis, synaptic loss, mitochondrial dysfunction, chronic neuroinflammation, and oxidative damage converge to degrade cognitive resilience (2, 3). Given the limited efficacy of current pharmacological therapies for age-related cognitive decline, there is growing interest in lifestyle-based strategies that tap endogenous repair and maintenance mechanisms (4).

Two modifiable factors, physical exercise and vitamin D, have been repeatedly implicated in supporting brain health. Exercise, particularly aerobic and resistance training, elevates key neurotrophic mediators such as brain-derived neurotrophic factor (BDNF), insulin-like growth factor 1 (IGF-1), and vascular endothelial growth factor (VEGF) (5–7). Meanwhile, it dampens pro-inflammatory signaling and enhancing mitochondrial and antioxidant capacity; these effects correlate with preserved hippocampal volume and improved cognitive performance in older adults (8–10). Vitamin D, acting through widely distributed vitamin D receptors (VDRs) and local activation within the brain, modulates neurotrophin expression, neurotransmitter systems, calcium homeostasis, and immune responses, with deficiency linked to increased risk of cognitive impairment and neurodegenerative pathology (11, 12). Preliminary multimodal trials and mechanistic studies, such as the SYNERGIC and PONDER frameworks and the combined exercise plus vitamin D work by Hoseini and colleagues, suggest that integrating these interventions may produce additive or synergistic neuroprotective effects, but a consolidated, aging-centered synthesis is lacking (13–15).

This narrative review therefore aims to bridge that gap by focusing explicitly on aging and neurodegeneration. We aim to:

1. Characterize how vitamin D and physical exercise individually influence neuroplasticity, neuroinflammation, and cognitive outcomes in aging and disease-relevant contexts.
2. Evaluate the evidence for their combined effects and articulate the biological mechanisms, shared and distinct, that could underlie synergy or augmentation.
3. By grounding translational claims in aging-specific and mechanistic primary data, we hope to clarify current evidence, expose critical uncertainties, and propose testable hypotheses for more targeted future interventions.

## 2 Exercise’s mechanisms of action as a modulator of neuroplasticity and cognitive function in aging and neurodegenerative conditions

Physiologic changes to the aging brain (i.e., cognitive aging) are complex and highly variable, resulting in difficulty projecting the trajectory of cognitive decline and identifying transition to pathologic states, such as MCI and dementia (16). Given the limited effectiveness of available treatments for dementia, promotion of a healthy brain is relevant. PE can promote cognitive brain health (defined as the ability to remember, learn, plan, concentrate, and maintain a clear, active mind) and counteract many effects of cognitive aging (17–19).

### 2.1 BDNF upregulation

One of the most consistently observed molecular changes triggered by exercise is the upregulation of BDNF, a neurotrophin essential for synaptic plasticity, neuronal survival, and cognitive function (20–22). Multiple studies across both animal models and human populations have shown that different durations, intensities, and types of exercise can enhance BDNF levels and activate downstream signaling pathways critical for brain health (23, 24).

In animal models, even short bouts of voluntary wheel running, ranging from a few hours to several days, can rapidly elevate BDNF expression in the hippocampus. For instance, one study found that 6 h of voluntary running significantly increased full-length BDNF mRNA in rats, with further elevation after 12 h. Interestingly, the response differed across BDNF exons: exon I increased rapidly, especially in the CA1 region, while exons III and IV showed minimal changes. The response was dose-dependent, with greater running distances correlating with higher BDNF levels (25). Another study has shown that moderate-intensity training in aging rats elevated BDNF and serotonin (5-HT) across multiple brain regions and was associated with improved memory and increased mature hippocampal neurons (7).

In human studies, aerobic training has been linked to both acute and chronic increases in serum BDNF. In a randomized trial, 3 months of endurance training in previously sedentary males significantly enhanced resting BDNF levels. The effect was most pronounced at rest, suggesting baseline neurotrophic support improves over time with consistent activity (26). Community-level interventions also support the value of exercise for aging cognition. A 12-week senior fitness program led to improvements in both strength and cognition, alongside elevated serum BDNF (27).

Complementary clinical studies in PD patients support these findings. In one trial, individuals with early-stage PD underwent an intensive 28-day rehabilitation program including daily aerobic exercise. Compared to controls, participants exhibited a significant rise in serum BDNF within the first 10 days, which was sustained through the end of the intervention. Functional improvements accompanied this neurobiological response, including better performance on the Unified Parkinson’s Disease Rating Scale (UPDRS) and enhanced balance and walking endurance (24). Further evidence from a UK-based pilot study examined how exercise intensity influences BDNF changes in early-to-mid-stage PD. Participants engaged in either moderate-intensity continuous training (MICT) or high-intensity interval training (HIIT), three times per week over 12 weeks. While acute sessions did not significantly alter BDNF levels, a notable increase was observed over time in the HIIT group, suggesting that sustained high-intensity exercise may be particularly effective in promoting neurotrophic support (28).

Collectively, these human trials suggest that regular exercise, particularly higher-intensity or structured programs, can enhance BDNF levels in populations vulnerable to cognitive decline and neurodegeneration. This neurobiological response may underlie some of the functional and cognitive improvements observed in clinical and experimental settings.

### 2.2 IGF-1 and VEGF pathways

IGF-1 is a conserved neurotrophic hormone important for brain development, neuronal survival, and synaptic remodeling (29, 30). Its signaling through pathways such as PI3K–Akt contributes to both neurogenesis and metabolic regulation (31). VEGF, while traditionally studied for angiogenesis, also promotes neurogenesis and supports vascular scaffolding necessary for synaptic remodeling (32).

A rodent study in which Rats received 4 weeks of treadmill training showed improved performance in spatial learning tasks compared to sedentary AD model animals. This improvement was accompanied by a restoration of hippocampal VEGF signaling, including increased mRNA expression of HIF-1α, VEGF, and VEGFR2, all of which had been suppressed by Aβ injection. These findings suggest that exercise enhances neurovascular function and cognitive resilience by stimulating VEGF-related pathways while dampening anti-angiogenic signaling (33). In a separate study using aged, sedentary rats, reduced levels of IGF-1, VEGF, and longevity-associated proteins like SIRT1 and SIRT3 were found in the substantia nigra, a brain region particularly vulnerable in Parkinson’s disease. These deficits were associated with an imbalance in the brain’s local renin–angiotensin system (RAS), marked by increased activation of pro-inflammatory Ang II/AT1R signaling and reduced activity in the protective Ang 1–7/Mas receptor axis. Remarkably, 4 weeks of treadmill running reversed these age-related changes, leading to higher IGF-1 and VEGF expression, upregulation of SIRT1 and SIRT3, and a shift toward a neuroprotective RAS profile. Thus, exercise may help prevent dopaminergic neuron loss in aging (34).

In humans, exercise interventions reliably increase IGF-1 levels. In a 16-week aquatic training study with elderly women (ages 68–80), moderate aerobic activity significantly increased IGF-1 and BDNF, accompanied by cognitive improvements compared to controls (35). Similarly, older men engaging in either resistance or endurance exercise showed post-session increases in serum IGF-1 and BDNF. The training effects did not significantly differ between modalities, suggesting both aerobic and anaerobic approaches are effective (36). However, peripheral IGF-1 levels do not always translate directly to brain outcomes. In a three-month trial with older adults (ages 60–77), treadmill exercise did not significantly change group-average IGF-1 levels, yet individual increases were associated with greater hippocampal volume and better verbal recall. This suggests that IGF-1 may function more as a permissive factor than a sole driver of neuroplasticity (37). In Parkinson’s disease, exercise may fail to induce expected VEGF responses. One study with task-oriented and aerobic training showed no significant changes in VEGF or IGF-1, suggesting that disease severity or short intervention duration might blunt neurotrophic adaptation. Additionally, peripheral levels may not always reflect central effects, especially in neurodegenerative contexts (38).

Taken together, these studies highlight that both IGF-1 and VEGF signaling are possibly involved exercise-induced benefits during aging. However, it is important to consider individual variability in IGF-1 and VEGF responses to exercise. Factors such as age, sex, baseline fitness level, genetic polymorphisms, and even vitamin D status may modulate the degree to which these growth factors are upregulated. For example, older adults or individuals with metabolic dysregulation may exhibit a blunted IGF-1 response compared to younger, healthier individuals (39). Recognizing this heterogeneity is crucial for understanding who may benefit most from exercise-based interventions and for translating these findings into personalized strategies for brain health.

### 2.3 Anti-inflammatory effects

Chronic inflammation is a hallmark of aging and contributes to cognitive decline (40). Physical exercise, particularly aerobic and resistance training, modulates both systemic and neural inflammation, potentially supporting cognition by reducing cytokine levels and enhancing neuroimmune balance (41).

In one study, aged rats (18 months old) underwent a 10-day treadmill training protocol, after which hippocampal cytokine levels were assessed. While levels of TNF-*α*, IL-1β, and IL-6 did not significantly differ between exercised and sedentary groups when measured individually, the ratios of pro- to anti-inflammatory markers (IL-1β/IL-10, IL-6/IL-10, TNF-α/IL-10) were significantly reduced in the exercise group. This indicates a shift toward an anti-inflammatory profile, suggesting that even short aerobic training can recalibrate the inflammatory environment in aging brains (42). Supporting this, another study using a D-galactose-induced aging model in rats demonstrated that regular swimming exercise led to significant neuroprotective effects. In aging rats that swam regularly, expression of inflammatory signaling molecules in the hippocampus was significantly downregulated compared to non-exercised aging counterparts. At the same time, key survival-related pathways, including AMPK/SIRT1/PGC-1α and IGF-1/PI3K/Akt, were upregulated, indicating improved mitochondrial function and neuronal resilience. Exercise also suppressed both Fas- and mitochondria-dependent apoptotic signaling, which were otherwise heightened in aged animals. Together, these effects suggest that physical activity can modulate both inflammatory and apoptotic mechanisms that contribute to cognitive decline with age (43).

In a 12-week moderate aerobic training study with older adults (ages 50–85), those with mild cognitive impairment (MCI) showed significant reductions in cortisol, IL-6, TNF-*α*, and CRP after intervention. These changes were accompanied by improvements in attention and executive function. The results suggest that lowering systemic inflammation through physical activity may help restore cognitive performance in at-risk individuals (44). A 16-week multimodal training program in both healthy elderly and individuals with MCI also reduced IL-6 and TNF-*α*, while increasing BDNF and improving cognition. Another three-month intervention involving exercise or exercise plus cognitive training found that both reduced IL-1β, IL-6, and p-tau181. Interestingly, the exercise-only group also showed a favorable Aβ42/Aβ40 ratio, an encouraging finding given the association of these biomarkers with Alzheimer’s disease (45). Resistance training, although studied less often in this context, has also demonstrated anti-inflammatory effects. In older women (around mean age of 83 years), 28 weeks of elastic band training elevated IL-10 (an anti-inflammatory cytokine) and stabilized TNF-*α* and CRP levels. This group also showed improvements in MMSE scores and physical fitness (46).

Thus, by reducing pro-inflammatory cytokines like IL-6 and TNF-α, exercise can lower microglial activation, support hippocampal neurogenesis, and improve synaptic remodeling. These immune shifts likely create a more permissive environment for plasticity and cognitive maintenance in aging.

### 2.4 Oxidative stress reduction

Oxidative stress increases with age and contributes to mitochondrial dysfunction and cognitive decline (47). Exercise improves antioxidant defense systems, both peripherally and within the brain (48, 49).

Animal studies have offer mechanistic insights into this subject. For instance, an investigation explored the effects of moderate treadmill exercise in middle-aged female Wistar rats over 15 weeks. The hippocampus of exercised rats showed significantly reduced ROS levels and protein carbonyl content compared to sedentary controls. Furthermore, exercise led to upregulation of key antioxidant enzymes such as superoxide dismutase-1 (SOD-1) and glutathione peroxidase (GPx). Enhanced activity of AMP-activated protein kinase (AMPK) and increased expression of PGC-1α, NRF-1, and mitochondrial transcription factor A (mtTFA) suggested that mitochondrial biogenesis was stimulated, improving cellular energy balance and reducing oxidative damage in the brain. This points to a systemic mitochondrial and antioxidant remodeling process driven by sustained physical activity (50). Another nine-week swimming program in young and middle-aged Wistar rats also demonstrated cognitive and oxidative benefits. Exercised rats showed improved memory and learning performance in avoidance tasks, accompanied by reduced protein carbonyl content in the brain. This reduction in oxidative protein damage was paralleled by an increase in the proteasome’s chymotrypsin-like activity, which plays a role in degrading oxidized proteins. The enzyme DT-diaphorase, another antioxidant marker, was elevated in young exercised rats, suggesting age-specific enzymatic adaptations to exercise (51).

In a 24-week human study (ages 65–95), aerobic training reduced oxidative stress markers (MDA, 8-OHdG) and inflammatory cytokines, while increasing total antioxidant capacity. Improvements in motor praxis and problem-solving correlated with these biochemical changes, supporting the cognitive relevance of redox balance in aging adults (52). Patients with Parkinson’s disease also show benefit. Over 12 weeks, aerobic and Tai Chi interventions increased antioxidant enzymes (e.g., GSH, catalase), with aerobic training producing greater effects. Cognitive scores measured by MMSE remained stable in the exercise groups but declined in controls. Furthermore, this study suggests that aerobic exercise may confer stronger neuroprotective effects than low-intensity alternatives like Tai Chi (53). Together, these findings suggest the possible role of exercise in reducing oxidative stress, thereby preserving cognitive function during aging. However, many studies report peripheral oxidative markers. Although useful, they do not always reflect CNS-specific changes. Future work should directly assess redox shifts in brain tissue or cerebrospinal fluid to confirm central effects.

### 2.5 Neurogenesis and synaptic plasticity

Exercise is increasingly recognized as a potent modulator of neurogenesis and synaptic plasticity, offering promising interventions for neurodegeneration, cognitive decline, and recovery from injury (50).

Evidence from animal studies shows that aging leads to a marked reduction in hippocampal neurogenesis, but exercise can partly counteract this decline. A mouse study evaluating neural stem/progenitor cells (NSCs) across different ages (3–21 months) demonstrated that the number of newborn neurons decreased by more than 95% by 9 months of age. While NSC counts were only mildly reduced, exercise attenuated this decline and enhanced neuronal lineage specification. However, exercise was unable to restore the dramatic reduction in NSC proliferation, the primary factor driving reduced neurogenesis with age. Interestingly, exercise significantly enhanced the maturation of newborn neurons, suggesting that its main benefit may lie in improving neuronal quality rather than quantity (54). The impact of exercise duration has also been investigated. In aged C57BL/6 J mice (19–23 months), access to a running wheel for 51 days, but not for 14 days, significantly increased hippocampal neurogenesis, as reflected by higher counts of doublecortin (DCX)-positive cells in the dentate gyrus. Long-term exercise also modulated the neuroimmune environment, increasing hippocampal levels of the anti-inflammatory cytokine IL-10, while both short- and long-term exercise reduced IL-6. These findings indicate that prolonged exercise exerts stronger effects on both neurogenesis and immune regulation in the aged brain (55).

In models of neurodegeneration, endurance exercise has been shown to engage multiple protective mechanisms. In a Parkinson’s disease mouse model induced with MPTP, regular endurance training reversed MPTP-induced motor deficits. Mechanistically, this was associated with increased BrdU-positive neurons, preservation of dopaminergic neurons (via elevated tyrosine hydroxylase and dopamine transporter levels), enhanced antioxidant defenses (e.g., catalase, GPX1/2, HO-1), and upregulation of autophagy markers such as LC3-II and Beclin-1. Collectively, these pathways supported both neurogenesis and neuronal survival, suggesting exercise acts through synergistic mechanisms (51).

## 3 Vitamin D mechanisms of action as a modulator of neuroplasticity and cognitive function in aging and neurodegenerative conditions

Vitamin D, traditionally recognized for its role in calcium homeostasis and bone metabolism, is now increasingly understood to function as a neurosteroid (56, 57). Its active form, 1,25-dihydroxyvitamin D₃, crosses the blood–brain barrier and binds to vitamin D receptors (VDRs) widely expressed in areas critical for cognition, such as the hippocampus, cortex, and substantia nigra (58, 59). Through these interactions, vitamin D influences neurogenesis, synaptic plasticity, and the expression of neurotrophic factors, including BDNF, essential for maintaining neural health and adaptability (60). Vitamin D deficiency (VDD) is notably common among older adults due to several age-related factors, such as reduced skin synthesis, limited sun exposure, and dietary insufficiencies (61). Estimates suggest that nearly 40–90% of elderly individuals may have suboptimal vitamin D levels, making it a significant public health concern in aging populations (62).

Studies have linked low vitamin D status to an increased risk of cognitive impairment, neurodegenerative diseases like Alzheimer’s and Parkinson’s, and mood disorders including depression (63, 64). Deficiency has been associated with accelerated brain aging, greater accumulation of amyloid plaques, and impaired neurotransmitter function (65, 66). Moreover, low vitamin D levels correlate with poor performance on memory and executive function tests (67).

### 3.1 VDRs and distribution

Active form of vitamin D, 1,25-dihydroxyvitamin D₃ (calcitriol), can cross the blood–brain barrier and interact with vitamin D receptors (VDRs), which are found in brain areas involved in cognition, including the hippocampus, prefrontal cortex, amygdala, and thalamus (68).

In both humans and rodents, VDRs are expressed throughout the lifespan, and the enzyme 1α-hydroxylase, which activates vitamin D, is also present in neurons and glial cells. These findings suggest that local vitamin D metabolism occurs within the brain, supporting region-specific actions. For instance, high expression of VDR and 1α-hydroxylase in the hippocampus and substantia nigra points to a potential influence on memory, motor control, and mood regulation (69).

In older adults, low circulating levels of vitamin D have been linked to increased lateral ventricle volume, a structural marker associated with brain atrophy and cognitive impairment. A study by Annweiler et al. (70) reported that individuals with vitamin D deficiency had significantly poorer performance on executive function tests, while Dhana et al. (71) found that intake of vitamin D was associated with slower rates of cognitive decline over time. Although these associations do not establish causality, they underscore the potential relevance of VDR signaling in age-related brain changes.

At the molecular level, VDR functions as a nuclear receptor, regulating transcription of genes related to neural development, plasticity, and immune signaling. It contains zinc-finger domains that bind to DNA, as well as a ligand-binding site that ensures specific activation by calcitriol (72). VDR activation affects numerous genes involved in antioxidant defenses, calcium balance, and neurotrophic factor expression (73, 74). In aging brains, these regulatory effects may help counteract inflammatory and oxidative processes that impair synaptic integrity.

Calcitriol also exerts rapid, non-genomic effects through the 1,25D₃-MARRS receptor (PDIA3), which is highly expressed in neurons, astrocytes, and endothelial cells (56, 75). PDIA3 activation has been linked to changes in BDNF and CREB signaling, factors essential for synaptic function and neurogenesis. However, its precise role in aging-related cognitive processes remains under investigation (76).

Taken together, the widespread expression of VDRs and vitamin D–metabolizing enzymes throughout the aging brain supports the plausibility of vitamin D as a neuromodulator in later life. While observational studies in humans show mixed results, several report structural and functional associations between low vitamin D levels and cognitive risk markers, particularly in executive and memory domains.

### 3.2 Regulation of neurotransmission and synaptic plasticity

Vitamin D appears to influence multiple neurotransmitter systems, including acetylcholine, dopamine, and serotonin, all of which are implicated in cognitive processes and are known to decline with age (77). By regulating the expression of enzymes and receptors involved in neurotransmitter synthesis and signaling, vitamin D may help preserve synaptic function during aging.

A study has been conducted on an animal model of AD, where LTP, a synaptic correlate of learning, was significantly impaired in rats infused with amyloid-*β* (Aβ) peptides. Interestingly, rats deprived of vitamin D exhibited even greater deficits in synaptic strength, as measured by reduced field excitatory postsynaptic potential amplitudes in the hippocampal CA3–CA1 circuit. In contrast, rats supplemented with the active form of vitamin D, 1,25(OH)₂D₃, showed restored LTP responses following tetanic stimulation, suggesting that vitamin D supports synaptic plasticity by preserving postsynaptic responsiveness under neurotoxic conditions (78). Complementary findings were reported in a separate study involving aging Fischer 344 rats fed diets with varying levels of vitamin D₃ over several months. Animals receiving high doses (10,000 IU/kg) performed significantly better in the Morris water maze reversal phase, an indicator of flexible learning, than those on medium or low vitamin D diets. Molecular analyses revealed that high-dose vitamin D upregulated hippocampal genes involved in synaptic communication, G-protein signaling, and basal neurotransmission. These transcriptional changes were accompanied by enhanced synaptic function, aligning with the observed behavioral improvements (79).

Aged rats subjected to D-galactose-induced brain aging and treated with high doses of vitamin D (1,000 and 10,000 IU/kg) for 8 weeks showed substantial reductions in hippocampal Aβ and MDA, a marker of oxidative stress, alongside increased glutathione (GSH). Improvements in memory, confirmed via the Barnes and novel object recognition tests, were paralleled by stronger expression of synaptic proteins such as PSD-95 and synaptophysin. These markers collectively point to vitamin D’s capacity to preserve synaptic integrity and function under stress-related conditions of aging (80).

Not all findings, however, are unanimously optimistic. A 10-week placebo-controlled trial involving older adults assessed whether 2,000 IU/day of vitamin D3 could modulate neuroplasticity via transcranial magnetic stimulation. Although vitamin D increased serum 25(OH)D from 46 to 81 nmol/L and led to modest improvements in muscle strength, it did not significantly enhance motor-evoked potentials (MEPs), short-interval intracortical inhibition (SICI), or serum BDNF compared to placebo. Despite the null results on neuroplasticity, the observed decrease in corticospinal excitability and inhibition in the treatment group might suggest improved synaptic efficiency, warranting further investigation over longer intervention periods or with higher doses (81). Together, these studies converge on a consistent theme: vitamin D supports neural communication and plasticity across the lifespan, particularly in regions such as the hippocampus that are vital for learning and memory. However, human data remain mixed, emphasizing the need for larger, longer-term studies to clarify the conditions under which vitamin D may optimize brain health.

### 3.3 Antioxidant and anti-inflammatory properties

Oxidative stress and chronic low-grade inflammation are central features of brain aging and contribute to neuronal damage, reduced synaptic plasticity, and cognitive decline (82, 83). Vitamin D has been shown to modulate both pathways through its actions on antioxidant enzymes and immune signaling molecules (84, 85).

In rodent models, aging is consistently linked to cognitive deficits and heightened inflammation. A study using 39 male F344 rats (20 months old vs. 6 months young) evaluated the impact of vitamin D (42 I. U./kg/day for 21 days via subcutaneous injection) on these age-related changes. Aged rats exhibited notable impairments in spatial memory and object recognition tasks. However, those receiving vitamin D supplementation showed significant improvements in both learning and memory performance, unlike the younger rats, where vitamin D had no noticeable effect (86). At the biochemical level, aging was associated with elevated levels of the pro-inflammatory cytokine IL-1β and reduced levels of the anti-inflammatory cytokine IL-10. Vitamin D reversed these trends in aged rats, indicating a reduction in neuroinflammation. Furthermore, aged rats treated with vitamin D demonstrated enhanced clearance of beta-amyloid (Aβ) and a corresponding decrease in amyloid load, a hallmark of neurodegenerative diseases like Alzheimer’s (86). These findings support vitamin D’s potential in moderating age-induced neurodegeneration via its anti-inflammatory and antioxidant actions.

Regarding human studies, a clinical study involving 52 participants (16 MCI, 11 very early AD, and 25 healthy controls), lymphocytes from patients with cognitive decline showed heightened vulnerability to oxidative stress when exposed to H₂O₂. After 6 months of vitamin D supplementation in those with low serum levels, MCI patients, but not early AD patients, exhibited reduced lymphocyte death and elevated plasma Aβ1-40 levels. Cognitive scores also improved at 18-month follow-up in MCI patients, suggesting that vitamin D’s protective role may be more effective at early stages of neurodegeneration (87). In another cohort of 140 elderly individuals (70 MCI cases vs. 70 controls), participants with MCI had significantly lower vitamin D levels (*p* < 0.05) and higher inflammatory cytokines, including IL-1β, IL-6, and TNF-*α*. White matter integrity was also compromised, particularly in brain regions such as the right temporal lobe and hippocampus. This correlation underscores the relationship between chronic inflammation, vitamin D deficiency, and structural brain changes in aging-related cognitive decline (88). Also, a 12-month double-blind RCT with 183 older adults with MCI assessed the effects of daily vitamin D supplementation (800 IU/day). Cognitive performance, as measured by full-scale IQ and subcomponents (e.g., vocabulary, digit span), improved significantly in the vitamin D group compared to placebo (*p* < 0.001). Moreover, telomere length was better preserved, and markers of oxidative stress (8-OXO-dG, OGG1 mRNA, P16INK4a mRNA) were markedly reduced. This suggests that vitamin D may attenuate cognitive decline via genomic stability and oxidative stress regulation (89).

By reducing inflammatory signaling and enhancing antioxidant defenses, vitamin D may help protect against structural and functional brain changes linked to aging. While direct central nervous system measures are lacking in most human studies, peripheral changes in inflammation and redox balance may reflect broader neuroprotective effects. Therefore, although findings from aging models are promising, variability in vitamin D dose, baseline status, and outcome measures limits the generalizability of results. More targeted studies in older populations using brain-specific biomarkers are needed to clarify the mechanistic pathways and therapeutic potential of vitamin D in cognitive aging.

## 4 Combined effects of exercise and vitamin D on brain health in aging and neurodegenerative conditions

### 4.1 Evidence from clinical trials in older adults

Multiple studies have explored the effects of combining vitamin D supplementation with exercise and other interventions to preserve or enhance cognitive function in older adults. While several mechanisms have been proposed for potential synergy, including modulation of neurotrophic factors, inflammatory control, and vascular health, few studies have systematically evaluated this interaction in clinical settings. However, a handful of recent trials have begun to fill this gap with mixed but informative results, shedding light on both the promise and limitations of these combined strategies.

One of the most comprehensive efforts to date is the SYNERGIC trial, a multi-site, double-masked, randomized controlled study that enrolled 175 adults aged 65–84 with MCI. The design was intentionally multidomain, assessing the independent and combined effects of aerobic-resistance exercise, computerized cognitive training, and vitamin D supplementation. Participants were randomized across five groups: a full intervention (all three elements), exercise with cognitive training (without vitamin D), exercise with vitamin D (without cognitive training), exercise alone, and a control group receiving only balance-toning activities and placebos. After 20 weeks, results showed that all active intervention arms involving aerobic-resistance exercise improved global cognitive function, as measured by the Alzheimer’s Disease Assessment Scale – Cognitive Subscale (ADAS-Cog-13), compared to control. Notably, combining exercise with computerized cognitive training resulted in further improvements over exercise alone. The most robust cognitive enhancement was seen in the full multidomain group, which included exercise, cognitive training, and vitamin D: this group showed a mean improvement of −2.64 points on the ADAS-Cog-13 compared to controls. However, when vitamin D was analyzed independently, comparing arms with and without supplementation, it did not appear to provide a statistically significant additive benefit (90).

While the primary outcome measures centered on cognitive performance, a related functional brain connectivity (FBC) study using the same trial cohort further examined whether combined interventions impacted neural network integration. FBC refers to the synchronous activation of brain regions over time and is known to decline in early cognitive impairment, particularly within the default mode network (DMN). In this sub-study, 120 participants underwent fMRI scanning before and after the 20-week intervention. Results showed that physical exercise, either alone or in combination with cognitive training and vitamin D, increased connectivity between key DMN regions, including the hippocampus and angular gyrus (FDR-adjusted *p* values < 0.05 across models). Although this enhanced connectivity did not correlate significantly with behavioral improvements in cognition or physical function, the findings suggest a neurobiological response to lifestyle intervention, even if functional implications remain unclear. Interestingly, the data showed that exercise alone may produce similar neural effects as more complex interventions, further emphasizing the potent influence of physical activity on the aging brain (91).

Another relevant trial, the PONDER study, aimed to assess the impact of a six-month multidomain intervention on cognition and physical health in older adults with subjective memory complaints. In this placebo-controlled RCT, 147 participants (mean age ≈ 70 years; 70% women) were randomized to receive either a combined program of resistance and aerobic exercise plus a daily supplement containing vitamin D (1,000 IU), omega-3 fatty acids, and protein, or a control condition involving flexibility training and a placebo supplement. Despite its robust design, the intervention did not lead to statistically significant improvements in cognitive performance (as assessed by CogState and Trail Making Test B-A) or physical function at either 6 or 12 months. However, the group receiving the active intervention showed a significant increase in lean muscle mass compared to controls (+0.72 kg; 95% CI: 0.26–1.19; *p* = 0.001), which may reflect improved physiological reserve and future resilience (92).

Taken together, these trials offer a nuanced picture of the interaction between exercise and vitamin D in aging populations. The SYNERGIC study demonstrates clear cognitive benefits from aerobic-resistance training and cognitive training, with only modest or unclear additional effects from vitamin D supplementation. The associated FBC analysis adds support for exercise-induced neural plasticity, while the PONDER trial illustrates the potential for body composition improvements without corresponding cognitive gains, possibly due to its broader supplement mix and different population characteristics (subjective vs. objective memory impairment).

These findings suggest the need for well-powered, aging-specific randomized controlled trials. While current evidence supports exercise as a cornerstone for cognitive health, the role of vitamin D in this context remains less conclusive. Future studies should explore whether synergy emerges in subgroups (e.g., those with baseline vitamin D deficiency, frailty, or low BDNF levels), and incorporate biomarkers or imaging data to clarify mechanistic pathways.

### 4.2 Mechanistic animal studies showing synergistic neurobiological effects

Cognitive impairment is a central feature of aging-related neurodegenerative conditions such as Alzheimer’s and Parkinson’s disease. Both vitamin D and physical exercise have been independently linked to improvements in cognitive outcomes through mechanisms such as neurotrophin upregulation, neurotransmitter regulation, and oxidative stress reduction. Recent animal studies using aging-related disease models have begun to assess whether these two interventions may interact synergistically to improve memory, learning, and neurocognitive biomarkers.

One such study used a lipopolysaccharide (LPS)-induced Alzheimer’s model in rats to evaluate the independent and combined effects of vitamin D supplementation and aerobic exercise on cognitive and biochemical outcomes. Fifty rats were assigned to five groups, including a control, Alzheimer model, vitamin D-only treatment, exercise-only treatment, and a combined vitamin D plus exercise group. Multiple markers were assessed in brain tissue, including acetylcholine esterase (AChE), brain-derived neurotrophic factor (BDNF), nerve growth factor (NGF), amyloid-*β*42, tau protein, and pro- and anti-inflammatory cytokines such as IL-6 and IL-10. The Alzheimer group demonstrated substantial deficits in multiple domains, including elevated levels of amyloid-β, tau proteins, IL-6, and malondialdehyde (MDA), a lipid peroxidation marker. These rats also showed decreased concentrations of BDNF, NGF, dopamine, AChE, and GSH (an endogenous antioxidant), as well as impairments in T-maze performance. However, both vitamin D and exercise individually improved several of these markers, and the combined treatment group showed the most significant improvements across nearly all domains.

Quantitatively, the combined group demonstrated a marked reduction in IL-6 and MDA levels, alongside increased BDNF, NGF, dopamine, and AChE expression in brain tissue. The T-maze test time, a functional index of spatial memory, was also significantly reduced in the combined group compared to either intervention alone. These data suggest a synergistic effect of vitamin D and exercise in reversing neuroinflammation and oxidative stress, as well as restoring cholinergic and dopaminergic signaling pathways crucial for memory function (93).

Another study examined the effects of exercise and vitamin D3 (VD3) supplementation in 6-hydroxydopamine (6-OHDA)-lesioned hemiparkinsonian rats, a model commonly used to simulate Parkinson’s disease-related neurodegeneration. Rats were divided into sham, lesion-only, and intervention groups (VD3 only, exercise only, and combined). The exercise protocol involved 30 min of treadmill activity daily for 21 days, initiated 24 h post-lesion. The researchers assessed motor function (rotarod, open field, and apomorphine-induced rotation tests), as well as biochemical markers like dopamine (DA), 3,4-dihydroxyphenylacetic acid (DOPAC), and tyrosine hydroxylase (TH) in striatal tissue. Markers of oxidative stress, (e.g., nitrite/nitrate, MDA, GSH) and receptor expression for vitamin D (VD3R) and dopamine transporter (DAT) were also evaluated. In 6-OHDA-lesioned rats, motor and cognitive performance were significantly impaired, and DA, TH, and DAT levels were markedly reduced. Treatment with vitamin D or exercise alone partially restored these deficits. However, the combined intervention led to the greatest improvement in dopaminergic function, with DA and DOPAC concentrations approaching levels seen in sham-operated controls. The group receiving both interventions also showed normalized TH and DAT expression, indicating a restoration of dopaminergic neurons. Moreover, oxidative stress markers were significantly reduced in the combined treatment group. Specifically, MDA levels were decreased, and GSH levels increased, supporting the view that the combination of exercise and vitamin D may mitigate oxidative damage in Parkinsonian neurodegeneration. Importantly, vitamin D receptor (VD3R) expression, which was downregulated in the lesioned brain, was restored by the combination of exercise and VD3. This implies that exercise may enhance the neurobiological response to vitamin D by increasing receptor availability or sensitivity, thereby strengthening its neuroprotective effects (94).

These findings align with the mechanistic concerns regarding the need for aging-specific animal models and biologically plausible explanations for any proposed synergy. Both studies provide strong support for a multi-modal approach, demonstrating that combining vitamin D and exercise yields greater improvements in cognitive markers, neurotrophic support, neurotransmitter restoration, and oxidative balance than either intervention alone. While results from preclinical models cannot be directly extrapolated to humans without caution, they offer a compelling rationale for future trials targeting early cognitive decline in older adults with combined lifestyle and nutritional interventions.

### 4.3 Observational insights and population-level findings

Beyond clinical trials and animal studies, large-scale observational data offer valuable insights into how vitamin D and physical activity may interact in everyday life to influence the biological aging process. Though causality cannot be inferred from such data, these findings can inform public health strategies and guide future intervention trials. Notably, population-level studies suggest that higher vitamin D levels and regular physical activity may synergistically contribute to healthier aging trajectories, including slower biological aging and better physical function.

A recent analysis using data from 18,738 participants of the National Health and Nutrition Examination Survey (NHANES) investigated how serum 25-hydroxyvitamin D [25(OH)D] levels and physical activity relate to Phenotypic Age Acceleration (PhenoAgeAccel), a biomarker-driven estimate of biological aging relative to chronological age. In multivariable models adjusted for demographic and health factors, individuals with both high vitamin D levels and adequate physical activity had significantly lower odds of accelerated aging (OR = 0.657; 95% CI: 0.549–0.787; *p* < 0.001). Among adults aged 65 and younger, this protective effect remained strong (OR = 0.663; 95% CI: 0.538–0.818; p < 0.001), and a multiplicative interaction was observed between vitamin D and PA (OR = 0.729; 95% CI: 0.542–0.979; *p* = 0.036), suggesting a synergistic relationship rather than independent effects alone. From a public health perspective, the implications were striking: 14.3% of accelerated aging could be attributed to low vitamin D, and 14.2% to physical inactivity, but their combined absence accounted for a 30.7% reduction in PhenoAgeAccel. Participants with both optimal vitamin D (>80.4 nmol/L) and adequate physical activity showed an average biological age reduction of 1.29 years compared to less active or deficient peers (*p* < 0.001). These findings offer epidemiological support for lifestyle-based interventions that target both movement and micronutrient sufficiency to delay age-related physiological decline (95).

In contrast, the DO-HEALTH randomized clinical trial, which enrolled over 2,150 older adults across Europe (mean age of 75 years), tested a combination of vitamin D3 supplementation (2000 IU/day), omega-3 fatty acids (1 g/day), and a strength-training program over a three-year period. The study used a robust 2 × 2 × 2 factorial design, allowing for analysis of individual and combined effects across eight intervention arms. The six primary outcomes included changes in blood pressure, physical performance (Short Physical Performance Battery), cognitive function (Montreal Cognitive Assessment), fracture rates, and infection incidence. Despite the trial’s large sample size and long follow-up, none of the interventions, alone or combined, produced statistically significant improvements in any of the six endpoints at 3 years. For example, the mean difference in systolic blood pressure for vitamin D vs. placebo was −0.8 mmHg, and cognitive performance (MoCA scores) remained unchanged across groups. Physical performance and fracture rates were also unaffected. Interestingly, there was a small but non-significant trend toward lower infection rates in the omega-3 groups, but this did not meet the trial’s threshold for statistical significance (*p* < 0.01) (96). These mixed results highlight the gap that often exists between observational associations and controlled intervention outcomes. While population-level data suggest that vitamin D and physical activity may work together to slow biological aging, large-scale RCTs like DO-HEALTH have yet to confirm these effects on functional or cognitive endpoints, particularly in already healthy, high-functioning older adults. Noteworthy, synergistic effects, while biologically plausible and supported in some settings, may not manifest uniformly in every population or under every protocol. One possible explanation is that baseline vitamin D levels and activity thresholds matter; DO-HEALTH participants were relatively healthy at enrollment, limiting the potential for detectable improvement. Moreover, variations in adherence, dose–response relationships, and duration of intervention may also influence outcomes. These nuances underscore the need for more targeted trials in vulnerable subgroups (e.g., frail older adults, those with early cognitive decline, or individuals with low baseline vitamin D).

**Figure 1:**
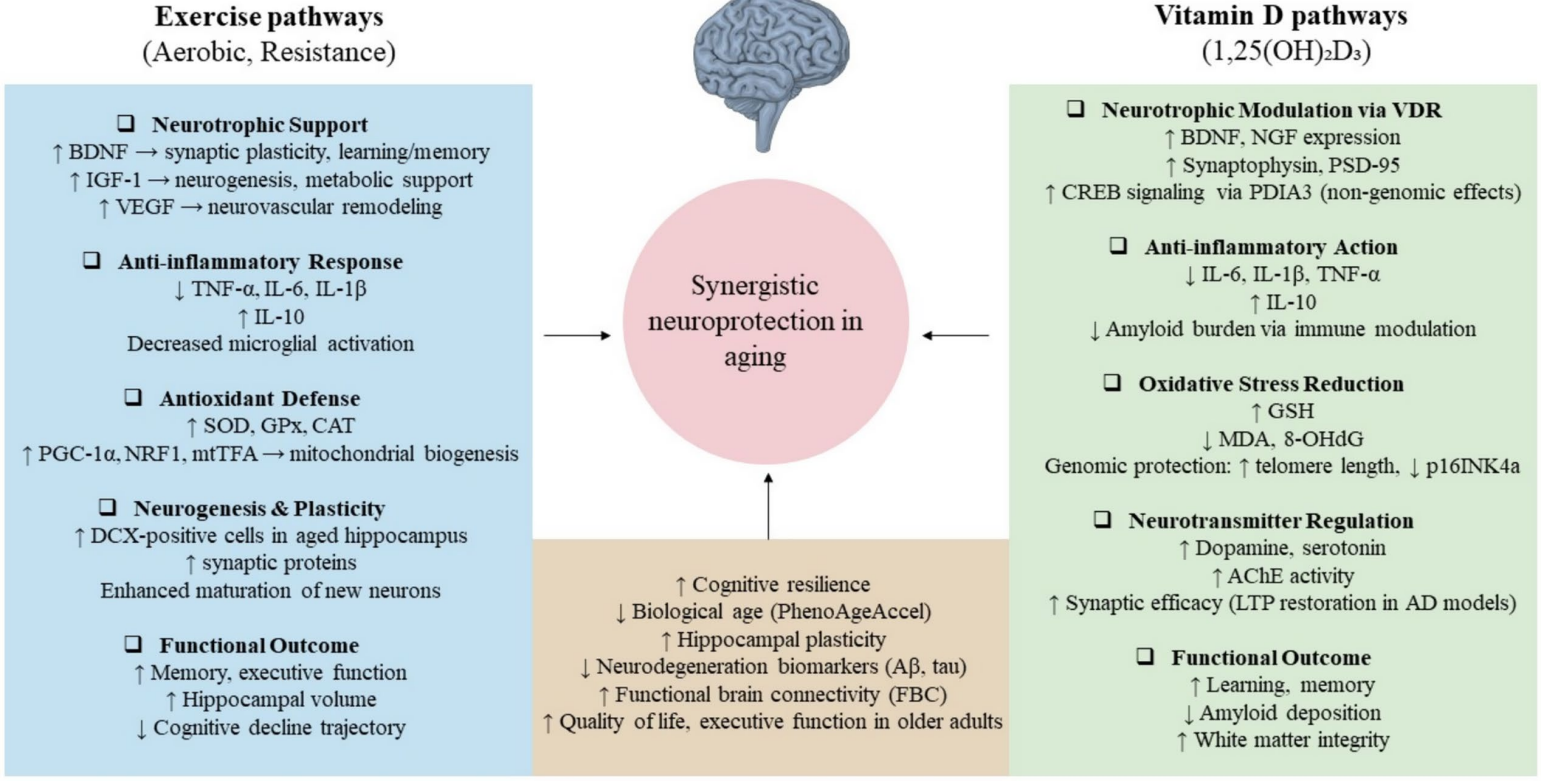
Converging mechanisms by which exercise and vitamin D may promote neuroprotection in aging. Exercise enhances neurotrophic signaling (e.g., BDNF, IGF-1, VEGF), reduces inflammation and oxidative stress, and supports neurogenesis. Vitamin D acts via genomic (VDR) and non-genomic (PDIA3) pathways to modulate neurotransmission, antioxidant responses, and neuroinflammation. Shared outcomes include improved cognitive function, hippocampal plasticity, and resilience to neurodegeneration. Together, these mechanisms suggest a plausible biological synergy in delaying cognitive aging.

In summary, observational studies strongly support the combined benefit of vitamin D and physical activity in reducing biological aging, but large RCTs have produced more conservative findings, emphasizing the complexity of translating population-level associations into clinical outcomes. Together, these studies suggest that vitamin D and exercise may exert complementary effects on aging biology, but their synergy is likely modulated by individual context, intervention intensity, and underlying risk status, as reviewers have rightly emphasized. Table 1 summarizes the key studies that evaluated the combined effects of exercise and vitamin D on cognitive and physical outcomes in aging populations. A conceptual schematic of converging pathways of exercise and vitamin D is also shown in Figure 1, highlighting shared biological targets implicated in aging-related cognitive decline.

**Table 1:** Summary of human and animal studies investigating the combined effects of vitamin D and exercise on aging-related outcomes.

Population/model	Design/intervention arms	Duration	Main outcomes measured	Key findings	Synergy observed	Aging relevance	Ref
Older adults with mild cognitive impairment (mean age 73.1)	5 arms: Exercise + Cognitive + Vitamin D, Exercise + Cognitive + Placebo, Exercise + Sham Cognitive + Vitamin D, Exercise + Sham Cognitive + Placebo, Control (stretching + placebo)	20-week intervention + 6-month outcomes	ADAS-Cog-13, ADAS-Cog-Plus	All exercise arms improved ADAS-Cog-13 vs. control (mean Δ = −1.79; *p* = 0.02); combo of exercise + cognitive training improved cognition more than exercise alone; Vitamin D had no independent effect	Partial (Exercise + cognitive synergistic; Vit D not effective)	Human aging (older adults with MCI)	(90)
Older adults with subjective memory impairment (mean age 70.2)	Multidomain: Exercise + Omega-3 + Vitamin D + Protein vs. placebo + flexibility/stretching control	6-month intervention + 6-month follow-up	Cognitive function (CogState, Trail-Making Test B-A), physical function, lean mass	No significant cognitive or functional gains; intervention increased lean mass (*Δ* = 0.72 kg, *p* = 0.001)	No	Human aging (older adults)	(92)
Older adults with mild cognitive impairment (mean age 73.9)	5 arms: Physical exercise, cognitive training, vitamin D, combinations, and control	20 weeks	Functional brain connectivity (FBC) via CONN toolbox; cognitive & physical function	FBC significantly increased with exercise alone, exercise + cognitive training, and full combination; no clear correlation with cognitive or physical outcomes	Partial (FBC improved; no behavioral synergy evident)	Human aging (older adults with MCI)	(91)
6-OHDA-lesioned hemiparkinsonian rats	Sham, 6-OHDA + VD3, 6-OHDA + exercise, 6-OHDA + VD3 + exercise	21 days	Behavioral tests (rotarod, open field), DA/DOPAC levels, oxidative stress (MDA, GSH), TH, DAT, VD3R expression	Combined VD3 + exercise improved behavior, increased DA/DOPAC, normalized TH/DAT expression, reduced oxidative stress more than either intervention alone	Yes	Animal aging model	(94)
18,738 adults (NHANES data, US)	Observational; stratified by 25(OH)D level & PA status	Cross-sectional (2007–2010 & 2015–2018)	Phenotypic Age Acceleration (PhenoAgeAccel)	High 25(OH)D + adequate PA reduced PhenoAgeAccel risk by 30.7%; combined group had 1.29 years lower biological age (p < 0.001)	Yes	Population-based biological aging marker	(95)
2,157 healthy older adults (≥70 years)	8-arm, 2 × 2 × 2 factorial RCT: Vitamin D3 (2000 IU), Omega-3 s (1 g), Exercise (strength training), alone and in combinations vs. placebo	3 years	BP, MoCA, SPPB, infections, nonvertebral fractures	No significant improvements in any primary outcomes with individual or combined interventions; only minor reduction in infection rate with omega-3 s (p = 0.02)	No	Human aging (healthy older adults)	(96)

## 5 Limitations

While this review highlights evidence supporting the potential synergy between exercise and vitamin D in promoting healthy brain aging, several limitations must be acknowledged.

First, a significant portion of the mechanistic data stems from preclinical animal models, which, while informative, cannot fully capture the complexity of human aging. Although recent studies have increasingly used age-relevant models such as lipopolysaccharide-induced Alzheimer’s disease and 6-hydroxydopamine-lesioned hemiparkinsonian rats, translation to clinical outcomes in older adults remains indirect. These models reflect neurodegenerative pathology but may not capture the multifactorial nature of aging in humans, particularly comorbidities, polypharmacy, and psychosocial factors.

Second, although we included recent human randomized controlled trials (e.g., SYNERGIC, DO-HEALTH, PONDER), the findings are mixed. For instance, the SYNERGIC study demonstrated improvements in cognitive function with combined aerobic-resistance exercise and cognitive training, but vitamin D alone showed no significant cognitive benefit. Similarly, in the PONDER and DO-HEALTH trials, no additive or synergistic effects were detected across cognitive, physical, or functional outcomes, despite adequate dosing and high adherence. These results suggest that while mechanistic synergy is plausible, clinical translation may be context-dependent, influenced by baseline health status, intervention intensity, duration, and population characteristics.

Third, there is a lack of uniformity in dosing, duration, and modality across studies. Vitamin D interventions varied widely, from daily to thrice-weekly regimens, ranging from 1,000 IU to 10,000 IU, making direct comparisons difficult. Exercise protocols also differed significantly in type (aerobic, resistance, multimodal), frequency, and supervision, limiting generalizability. This heterogeneity, noted by reviewers, complicates conclusions about the “optimal” combination strategy and may partially explain the variability in observed outcomes.

Fourth, some included studies utilized composite interventions involving additional factors such as omega-3 supplementation or cognitive training. While this reflects real-world multidomain strategies, it also introduces confounding, making it difficult to isolate the specific contribution of vitamin D or exercise to observed effects.

Fifth, there remains a paucity of studies explicitly designed to test for synergistic vs. additive interactions between vitamin D and exercise. Most trials assess main effects independently or in factorial designs without statistical modeling for interaction effects. The lack of such targeted designs limits the strength of conclusions regarding true synergy, as highlighted by reviewers.

**Figure 2:**
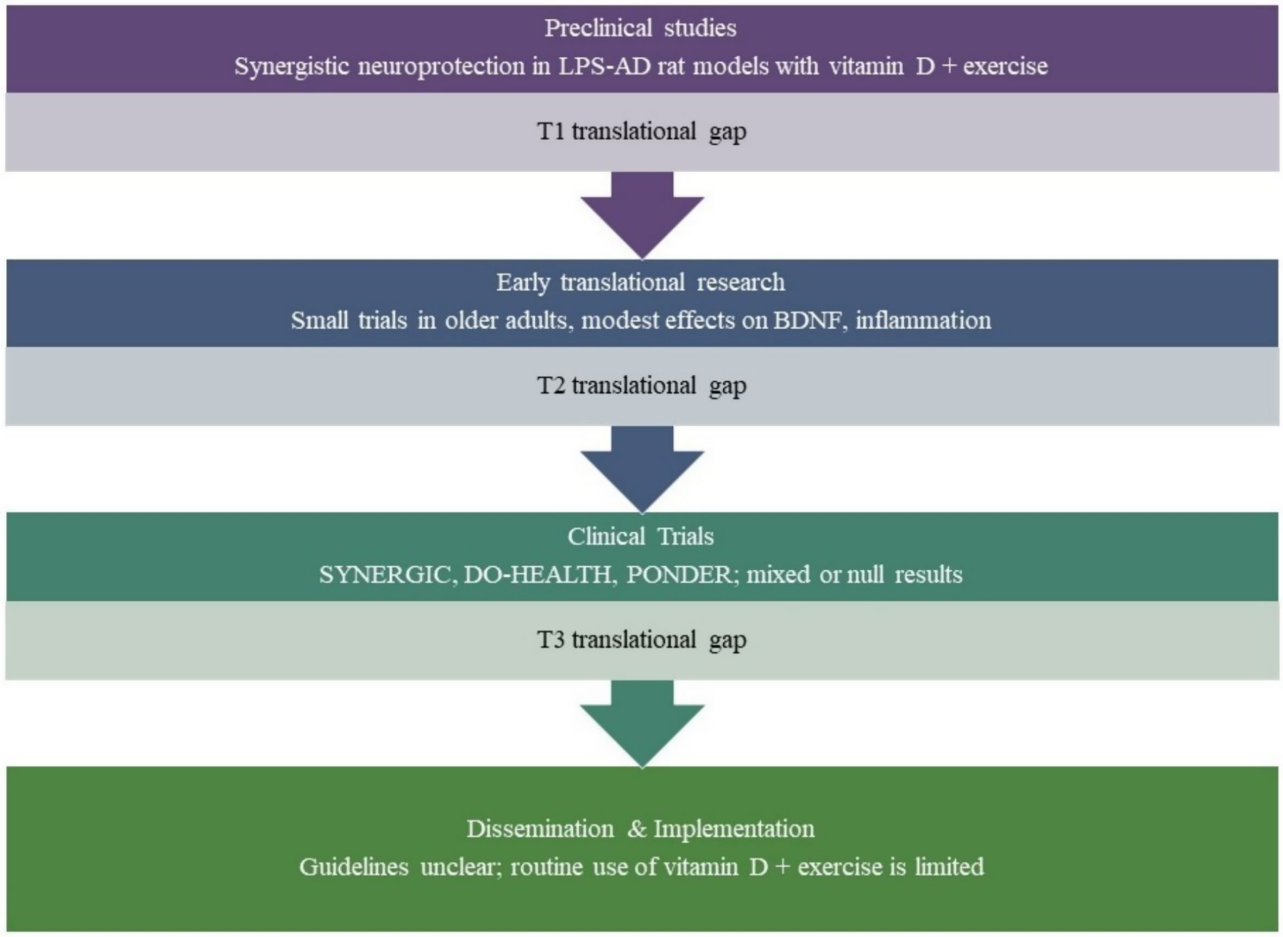
Conceptual model of translational gaps in vitamin D and exercise research for brain aging. This figure illustrates the progression of evidence from preclinical studies to clinical implementation, highlighting three key translational gaps (T1–T3). While synergistic effects between vitamin D and exercise are supported in rodent aging models (e.g., LPS-AD, 6-OHDA), small-scale human trials show only modest changes in biomarkers like BDNF and inflammatory cytokines. Large clinical trials such as SYNERGIC, DO-HEALTH, and PONDER report mixed or null results, and real-world guidelines rarely integrate combined interventions. This underscores the need for targeted trials in at-risk aging populations using standardized protocols and mechanistic endpoints.

Finally, most large-scale trials to date have been conducted in relatively healthy, high-functioning older adults, potentially underestimating the benefit of these interventions in vulnerable or low-functioning populations, those who may benefit most from preventive strategies. Additional research is needed in more diverse cohorts, including those with frailty, low baseline vitamin D status, or limited physical activity. As illustrated in Figure 2, substantial translational gaps remain between promising preclinical synergy and implementation in human aging contexts.

## 6 Future directions and conclusions

Emerging evidence from both clinical and preclinical studies suggests that exercise and vitamin D may exert complementary effects on brain aging, particularly in domains such as cognitive function, neuroprotection, and systemic aging markers. However, the strength and consistency of these effects, especially their synergy, remain uncertain. While several recent trials (e.g., SYNERGIC, PONDER, DO-HEALTH) have attempted to examine multidomain interventions in older adults, most have yielded modest or inconsistent outcomes, particularly regarding vitamin D’s independent or additive benefits. Moreover, mechanistic studies offer promising pathways, such as neurotrophin signaling, oxidative stress modulation, and inflammatory regulation, but are still primarily grounded in animal models.

Future research should focus on well-powered, targeted clinical trials specifically designed to test synergistic interactions, not just additive effects, between vitamin D and structured exercise. This requires careful selection of populations who are most likely to benefit, such as older adults with low serum 25(OH)D levels, sedentary lifestyles, or mild cognitive impairment, as well as standardized intervention protocols that allow clearer interpretation. Investigating dose–response relationships, optimizing intervention durations, and tracking longitudinal cognitive and functional outcomes will be critical. It is also essential to integrate biological markers (e.g., BDNF, IGF-1, VEGF, inflammatory cytokines) and neuroimaging measures (e.g., functional connectivity, hippocampal volume) into future studies, to better understand underlying mechanisms and personalize interventions. In addition, future trials should avoid overly complex combinations of multiple interventions (e.g., including omega-3 s or protein) unless specifically designed to disentangle their effects. In conclusion, although the potential for synergy between exercise and vitamin D in promoting healthy brain aging is biologically plausible and supported by select preclinical and epidemiological findings, current clinical evidence remains inconclusive. Well-designed studies targeting high-risk older adults and incorporating mechanistic and behavioral endpoints are needed to determine whether combining these two accessible, low-cost interventions can meaningfully delay or prevent age-related cognitive and functional decline.
